# Coproduction and health: Public and clinicians’ perceptions of the barriers and facilitators

**DOI:** 10.1111/hex.12834

**Published:** 2018-10-05

**Authors:** Daniella M. Holland‐Hart, Samia M. Addis, Adrian Edwards, Joyce E. Kenkre, Fiona Wood

**Affiliations:** ^1^ Division of Population Medicine School of Medicine Cardiff University Cardiff UK; ^2^ Faculty of Life Sciences and Education University of South Wales Pontypridd UK

## Abstract

**Background:**

Coproduction is an approach increasingly recognized across public services internationally. However, awareness of the term and the barriers and facilitators to its implementation in the NHS are not widely understood. This study examines clinician and public perceptions of coproduction within the context of the Prudent Healthcare initiative.

**Objectives:**

To provide insights into how coproduction is viewed by clinicians and the public and identify perceived barriers and facilitators to its implementation.

**Design:**

Using qualitative research methods, interviews were conducted with the public (n = 40) and clinicians (n = 40). Five focus groups were also conducted with the public (n = 45) and six focus groups with clinicians (n = 26). The COM‐B model was used to analyse the data; key domains include Capability, Opportunity and Motivation.

**Setting:**

This is an all‐Wales study, involving six Health Boards, an NHS trust and community and patient groups.

**Results:**

Key barriers relating to Capability include lack of awareness of the term coproduction and inadequate communication between clinicians and citizens. Opportunity‐centred barriers include service and time constraints. Conversely, facilitators included utilizing partnerships with community organizations. Motivation‐related barriers included preconceptions about patients’ limitations to coproduce.

**Conclusions:**

There were broadly positive perceptions among participants regarding coproduction, despite initial unfamiliarity with the term. Despite study limitations including underrepresentation of employed public participants and junior doctors, our analysis may assist researchers and policymakers who are designing, implementing and evaluating interventions to promote coproduction.

## 1 INTRODUCTION

Coproduction is an approach which emphasizes collaboration between service providers and citizens The term was originally popularized by Ostrom^1^ and further developed by Cahn.^2^ Definitions of coproduction vary;^3^, ^4^ however, most agree that it aims to promote the democratization of decisions made between citizens and service providers, working together to develop citizen‐centred outcomes. The New Economics Foundation described coproduction as a “means of delivering public services in an equal and reciprocal relationship between professionals, people using services, their families and their neighbours.”^5^

It is recognized that coproduction requires citizens to play an active role in their health care.^6^ Coproduction encourages citizens to participate as fully as possible at different levels and according to their needs and capacity.^7^ This includes citizens contributing to coproduction on an individual level, as well as in the design and delivery of services.^8^ However, lack of citizen coaching and opportunities to participate effectively can limit system‐level coproduction and tends to constrain it as a means to engage in individual patient‐level interactions.^9^

A more active and patient‐centred relationship between citizens and health‐care services has been increasingly championed by researchers and policymakers worldwide.^10^, ^11^, ^12^ Coproduction has increasingly featured as a core approach in public sector initiatives including examples cited by Co‐Create in Canada;^13^ the Organisation for Economic Cooperation and Development (OCED);^14^ and the National Endowment for Science Technology and Arts (NESTA)^15^ in the United Kingdom. Within this movement, there has been a focus on coproduction within health strategies, including the Scottish Government's Realistic Medicine,^10^ and across NHS England.^16^ In 2015, the Welsh Government's Prudent Healthcare initiative outlined the need to ensure the sustainability of the NHS against a background of constrained funding and increasing demand. A core principle of Prudent Healthcare was coproduction.

Although there has been policy energy invested in supporting health‐care services in Wales to adopt coproduction through the Prudent Healthcare initiative, there has been no prior assessment of public and clinician understanding and attitudes to coproduction through this initiative. Indeed, it was of particular interest to explore the views from those who expressed scepticism towards its purpose. This study aimed to understand how coproduction is viewed through the lens of the Prudent Healthcare initiative and to provide insights into facilitators and barriers to its implementation that were perceived by clinicians and the public. The focus was on the definition by the Bevan Commission,^17^ which is the basis of the Prudent Healthcare principle:

Co‐production refers to a way of working whereby decision makers and citizens, or service providers and users, work together to create and deliver services. This includes consideration of broader social, economic and cultural issues to avoid unnecessary medical and therapeutic interventions to resolve health care needs. (Bevan Commission, 2015) 17

## 2 METHODS

Semi‐structured interviews and focus groups were carried out with the public, patients and clinicians across Wales. The data were used to understand how coproduction was understood by clinicians and members of the public and how receptive they were to the concept, and also to identify barriers and facilitators to its implementation.

### 2.1 Participants

Purposive sampling was used to ensure diversity in age, gender and geographical area. We devised a sampling strategy to capture perspectives from members of the public who had varying health‐care experiences and access to services. We therefore included both men and women with long‐term conditions who were likely to have ongoing contact with health services; people with recent experience of using emergency health services; and parents of young children. We also speculated that people from different socio‐economic backgrounds might adopt different perspectives on coproduction, so we ensured we had representation of men and women from areas of low and high socio‐economic deprivation (determined by the Welsh Index of Multiple Deprivation).^18^ Public participants were divided into five subsamples:

1. Patients with long‐term conditions
2. Patients who have recently accessed emergency care
3. Parents of young children
4. Members of the public from areas of high socio‐economic deprivation
5. Members of the public from areas of low socio‐economic deprivation.

For our clinician sample, our aim was to ensure representation from throughout Wales. The sample included participants from a variety of clinical backgrounds in the NHS including primary, secondary and emergency care settings and with varying grades and seniority.

### 2.2 Recruitment

Public participants were recruited through community groups (eg parent and toddler and Communities First groups), as well as through general practice surgeries. All participants were aged over 18 years. A member of the research team contacted various community groups to discuss the project and negotiate an opportunity to attend a session to provide information about the study to the group's membership. Three GP practices from across Wales agreed to help identify patients with long‐term conditions and patients who had recently experienced out‐of‐hours care. GPs invited patients who fulfilled the criteria to discuss the study with a researcher.

We recruited clinicians through six Health Boards and an NHS trust in Wales. Potential participants were approached via health board or trust partners or via professional organizations such as the Royal College of Nursing. Snowball sampling was also used.

Five public and patient involvement (PPI) representatives were recruited to contribute to early study documents, aid recruitment, comment on findings and assist with dissemination to lay audiences.

Interested potential participants were provided with an information pack outlining the purpose of the study and what was requested of them. If they agreed to participate, a time and place was arranged for an interview or a focus group. All participants provided written consent before participation.

### 2.3 Ethical approval

Ethical approval for the study was sought and granted (ref: 15/YH/0545). NHS R&D approval was given for recruiting of clinicians and recruiting of patients in primary care settings.

### 2.4 Data collection

Interviews typically were undertaken at the participants’ home or in community venues or, in the case of clinicians, at their place of work. Focus groups typically were undertaken in a community centre or on university or NHS premises. The interviews and focus groups were audio‐recorded, with the permission of the participants, transcribed and anonymized.

Following the interview or focus group, the researchers also collected some basic demographic data about the participants in order to fully describe the sample.

### 2.5 Theoretical framework

Our study was guided by the Behaviour Change Wheel (BCW), a framework that enables the systematic development of interventions for supporting behaviour change, in this case public and clinicians’ attitudes to coproduction. The Behaviour Change Wheel is underpinned by the COM‐B model which has three interacting conditions for behaviour change to occur: Capability (eg knowledge, cognitive abilities), Opportunity (eg access, cultural norms) and Motivation (eg beliefs, values).^19^

### 2.6 Data analysis

Using the COM‐B model framework, data analysis was supported by the NVIVO software program. Data analysis followed a framework approach^20^ where data were reread and common ideas and patterns emerging from interviews and focus groups were identified and coded by one author (DHH), grouped into subthemes and further abstracted to form broad themes, using both deductive (researcher‐driven) and inductive (response‐based) methods. Following this, themes were reviewed, refined and classified using the components of the COM‐B model^19^ in an iterative process with refinement from coauthors.

## 3 RESULTS

Participants included 85 members of the public; 40 took part in face‐to‐face interviews and 45 in five focus groups (Table 1). Participants could belong to more than one sample category (eg parents of young children living in an area of low socio‐economic deprivation), but the table describes the sample characteristics on the basis of which they were recruited.

**Table 1:** Public and patient participant characteristics

Participant characteristic	Number of participants (%)
Sample	
Patients with long‐term conditions	15 (18)
Patients who sought emergency care	16 (19)
Parents of young children	18 (21)
Public from areas high socio‐economic deprivation	19 (22)
Public from affluent areas	17 (20)
Health board area of home residence	
Aneurin Bevan	20 (24)
Abertawe Bro Morgannwg	14 (16)
Betsi Cadwaladr	12 (14)
Cardiff & Vale	10 (12)
Cwm Taf	15 (18
Hywel Dda	12 (14)
Powys	2 (2)
Gender	
Male	37 (44
Female	48 (56)
Age group	
18‐25	10 (12)
26‐35	18 (21)
36‐45	14 (16)
46‐55	13 (15)
56‐65	9 (11)
66‐75	15 (18)
75+	6 (7)
Disability	
No disability	47 (55)
Disability	36 (43)
Prefer not to say	2 (2)
Employment status	
Carer	4 (5)
Employed	30 (35)
Retired	21 (25)
Unemployed	18 (21)
Volunteer	4 (5)
Missing data	8 (9)

Sixty‐six clinicians were recruited throughout Wales: general practitioners (GPs), nurses, hospital doctors, midwives, a pharmacist, a dentist, paramedics and allied health professionals such physiotherapists. Forty took part in face‐to‐face interviews, and 26 took part in six focus groups (Table 2).

**Table 2:** Clinician participant characteristics

Participant characteristic	Number of participants (%)
Service setting	
Primary care	23 (35)
Secondary care	28 (42)
Emergency and out‐of‐hours care	15 (23)
Health board or trust	
Aneurin Bevan	8 (12)
Abertawe Bro Morgannwg	8 (12)
Betsi Cadwaladr	6 (9)
Cardiff & Vale	22 (33)
Cwm Taf	15 (23)
Hywel Dda	2 (3)
Welsh Ambulance Service Trust	5 (8)
Gender	
Male	27 (41)
Female	39 (59)
Professional background	
Allied health professional	11 (16)
Dentist	1 (1.5)
GP	13 (20)
Hospital doctor	10 (15)
Midwife	3 (5)
Nurse	20 (30)
Paramedic	5 (8)
Pharmacist	2 (3)
Missing data	1 (1.5)
Years since qualification	
Less than one year	2 (3)
1‐2 y	2 (3)
3‐9 y	11 (16)
10‐14 ys	7 (11)
15+	38 (58)
Missing data	6 (9)

Results are presented in line with the COM‐B model's Capability, Opportunity and Motivation and include the barriers and facilitators of coproduction that participants identified within the context of Prudent Healthcare (Table 3).

**Table 3:** Results: barriers and facilitators to coproduction

COM‐B Theme	Barriers	Facilitators
Capability		
Physical capabilities	Poor physical and mental health	Support and advocacy
	Age (perceptions)	Bespoke advice
Psychological capabilities	Lack of Knowledge of coproduction and how to apply it	Increased publicity, education and training for citizens and staff
	Inadequate communication skills	Training in communication skills
	Lack of patient empowerment and self‐efficacy	Working with multidisciplinary teams
	Insufficient information and power sharing with patients	Change in cultural perceptions and practices
Opportunity		
Physical opportunities	Allocation of appointment time	Restructure time allocation in services
Social opportunities	Socio‐economic inequalities	Partnerships with community groups
Motivation		
Reflective motivation	Negative experiences of inclusion	Positive experiences of inclusion
Automatic motivation	Preconceptions of patient or service limitations	Challenging preconceptions about practice

### 3.1 Capability

Participants recognized that the capability of each citizen to participate in coproduction varies. Overall physical health including mental health and psychological factors such as knowledge and health literacy, empowerment, self‐efficacy and communication skills were highlighted as facilitators and barriers.

### 3.2 Physical capabilities

While participants generally agreed that citizens should take a certain amount of shared responsibility over their own health, they also recognized that facilitators such as appropriate support were required for individuals to be able to fully participate: “I'm all for people taking at least some responsibility for their own health, given the right or appropriate information” (public participant from an affluent area).

It was acknowledged that the physical inequalities can hinder people's capacity to play an active role in their health and that coproduction may be too challenging for some. Poor health, especially mental health, was recognized as a significant barrier to effective coproduction. Some clinicians recognized that extra support and advocacy should be provided in such cases, to enable patients to contribute to their full capacity, no matter how limited that may be: “There's always the danger that you could exclude people who don't feel that they have a voice, either through physical or mental abilities… what we need is far greater advocacy” (primary care clinician, GP).

Many participants perceived that age and educational background contributed to whether someone would be able and willing to participate in coproduction: “I think it's probably the younger more educated but I think the older generation is still of that generation that the doctor knows best and whatever they tell me I will accept” (secondary care clinician, midwife). However, there was little evidence from the public participants that older people would not want to, or be able to, coproduce, but rather that the assumption itself is a barrier and may need to be challenged.

### 3.3 Psychological capabilities

#### 3.3.1 Health literacy and knowledge

Relatively few public participants and only about a third of clinicians recalled any prior knowledge of the term coproduction. Some expressed scepticism, including referring to it as a “buzzword.” Despite this, participants were broadly supportive of the concept, once it had been explained to them: “I had to ask what co‐production was and to me it was just something that made sense… It should have always been there” (patient with a long‐term condition).

Participants from both clinician and public groups discussed coproduction mainly in relation to one‐to‐one interactions between clinicians and patients. However, some participants recognized that coproduction also involves citizens consciously contributing to the design and transformation of services. The term was interpreted in varying ways even after explanation. Confusion was expressed over what the concept actually meant: “It's not very clear to me what the role of public and patients is in the whole thing” (secondary care clinician, allied health professional). Despite this, a number of clinicians claimed that core elements of coproduction have been long established within the NHS and provided examples of coproduction: “Involving the patients as partners is very much centre to what we do” (primary care clinician, GP).

Clinicians and members of the public felt there was a need to establish a more informed citizenry by introducing health education into schools and at a wider community level, in order to facilitate coproduction. Likewise, some participants also recalled or suggested using community groups, NHS professionals and the media to help inform citizens about issues related to their health. Participants argued that patients who were better informed about health choices, treatments and health services were generally more autonomous and would experience better health outcomes: “A well‐educated group of patients not only achieves more through engagement and empowerment in their own health but they actually make better use of NHS resources because they're accessing the right professionals about the right query” (secondary care clinician, pharmacist).

#### 3.3.2 Communication skills

Participants reflected that improved communication between patients and professionals was central to the successful enactment of coproduction.

Members of the public emphasized a need for clinicians to consider the patient's individual needs and circumstances, as patient dissatisfaction and disengagement were often caused by incomplete or inadequate communication. Some patients reported having requested information to be communicated in more accessible formats: “With some of the jargon they've used, we've had it broken down to us, so now we're not afraid to ask questions and they put it in layman's terms so we can all understand” (public participant from an area of higher socio‐economic deprivation).

Being listened to by clinicians was equally important as receiving information, according to some participants: “Listening to the patients and definitely seeing what they want and then the patients maybe meeting up with GPs and working together about how they want it to be” (public participant from area of high socio‐economic deprivation).

According to a wide range of participants, clinicians need to be trained appropriately to communicate with and empower patients through coproduction: “That is dependent on health professionals having been trained to practice like that to start with and patients being encouraged to not just do as they're told” (patient who accessed emergency care). Ensuring that coproduction is part of the medical, nursing and allied health professionals’ curriculum to assimilate it as part of professional culture was considered potentially beneficial.

#### 3.3.3 Empowerment and self‐efficacy

Patient involvement was perceived by some participants as being central to service effectiveness: “I think patients are crucial to the development of services” (secondary care clinician, pharmacist). Among participants there was also a general consensus that the public had the right to know about their health and make decisions, as equal partners. However, participants conveyed contrasting views regarding the extent to which this could, and should, be achieved in practice: “Treating people as intelligent adults in charge of their own treatment, their own destiny. I think it's a positive thing but I think it might take a long time for that to really slowly get through” (parent).

Positive attitudes towards patient involvement was particularly evident among allied health professionals as well as clinicians who worked in multidisciplinary teams: “There's a lot less ‘yes doctor I'll do that’ you don't hear that anymore… they have as important a voice as anyone in their decisions made about their healthcare” (secondary care clinician, physiotherapist).

Some clinicians also felt that despite their good intentions to equalize power within the relationship, ultimately the balance of power may lean towards the practitioner: “You can't have equal partnerships where you've got an imbalance of power. In fairness we have the power. They're coming to us to ask us for treatment, we get the last say in whether or not they get that” (primary care clinician, nurse, focus group). This view of patient involvement was echoed in the views of some members of the public who were more sceptical about its application: “Working alongside the patient to find together a shared solution, I'm not sure that happens very often actually and I'm not sure it's the expectation of many patients” (public participant from area of high socio‐economic deprivation).

Although the participants recognized the potential benefits of greater empowerment, they reported varying levels of self‐efficacy. Some expressed concerns over of their capacity to be heard or to challenge the system, while others felt that some people would abuse the system “people will be kicking off and demanding to get treatment” (focus group public participant from an area of high socio‐economic deprivation).

Apprehensions about power imbalances were also conveyed by clinicians who felt increasing pressure to ensure patients are happy with what is offered to them: “Patients can be more demanding as a result and this can lead to wasted appointments as they demand to be seen more often” (primary care clinician, GP). Thus, participants’ highlighted tensions between patient empowerment and its practical delivery within an NHS that is struggling with resource constraints.

#### 3.3.4 Sharing information and decisions

Clinical and public participants largely felt that clinicians’ willingness to share and discuss information, including diagnoses and treatment choices, with patients was fundamental to successful coproduction. A number of public participants recalled positive experiences of being involved in information sharing and decision making. Despite positive accounts, a reluctance on the part of some practitioners to share information was noted by other clinicians: “We still have a culture where some clinicians don't feel that they can share the patient's records with them and discuss their pathways of care, or if the patients have got a concern there's still some trepidation about sharing clinical information” (secondary care clinician, midwife).

It was acknowledged that patients do not always have the opportunity to participate in decision making, as ultimately, responsibility for health care still rests with clinicians. Furthermore, clinicians acknowledged that not all practitioners practised patient‐centred medicine.

### 3.4 Opportunities

#### 3.4.1 Physical opportunities

The most significant service‐based barrier to coproduction reported by participants was time limitation: “I think time is really key, we're so pressurised for time seeing patients and the volume that want to be seen” (primary care clinician, GP).

Members of the public recognized that clinicians had to deal with competing priorities, as well as externally imposed targets which would reduce the time available to coproducing health care. One patient reflected on this service‐level barrier from a clinician's perceptive: “It's that I either spend the time that I would like with this patient and then I'm in trouble because I haven't met my target, or I meet my target but then I'm not giving that person the service they deserve” (patient with long‐term condition).

Due to what was perceived as being a time‐intensive process, some participants expressed scepticism about the resource effectiveness of coproduction. This emphasizes a need for further discussions between the public and clinicians regarding the purpose and benefits of coproduction. Underlying financial and practical limitations were perceived as impeding coproduction, as one parent questioned how patients’ requests are prioritized: “Does one person have the right to choose much more expensive treatment that enables them to do this and another person something else?” (parent).

#### 3.4.2 Social opportunities

Community‐based support was perceived to be an intrinsic element of the delivery of coproduction. This was particularly applicable where citizens faced health and social inequalities, as health‐based support groups and health advocates regularly acted as facilitators of coproduction. Some clinicians stated that they worked with local groups to provide bespoke patient‐centred health‐care provision within community settings: “If I go and run something in [community centre] no one will come, if you run it with the community group, with an interpreter, an advocate for that community, hundreds of people will turn up and they're all really interested” (secondary care clinician, allied health professional). Utilizing preexisting networks and building relationships with community‐based organizations were key to enabling coproduction at a community level, especially among those who face greater levels of socio‐economic deprivation or isolation.

### 3.5 Motivation

Clinicians and members of the public highlighted the importance of reflective motivations when considering the extent to which coproduction is achievable in practice. For some, active participation was a very positive experience: “Everyone loves taking part, especially in their own healthcare, they love feeling involved” (member of the public from area of high socio‐economic deprivation). Others argued that certain patients would not want to be involved in health‐care decisions: “I find that some patients you try and give them options, you try involve them in decision making and they don't want to be involved” (primary care clinician, GP).

It was also perceived that automatic motivations such as assumptions or preconceptions from either party could prevent coproduction. Examples from clinicians included the following: “Patients come with preconceptions and it used to be the preconceptions from a grandmother, or an aunty… these days they come with preconceptions they've picked up from the internet, from the media and that's often fed by political agendas” (primary care clinician, GP).

There was also recognition that patient demands, fuelled by emotions combined with financial concerns, can complicate the implementation of coproduction: “You don't want to be spending lots of money doing what the patient wants, if all the research and everything the doctor who has the knowledge is saying do something different… especially if you're talking about emotive stuff” (member of the public from area of low socio‐economic deprivation).

## 4 DISCUSSION

### 4.1 Principal findings

Coproduction has been described as a cornerstone of public policy reform^21^ and a potential vehicle through which to deliver effective public services in resource‐lean economies. Using the COM‐B model, a wide range of facilitators and barriers, seen to influence public and clinicians’ capability, opportunity and motivation to engage in coproduction, was identified.

The most commonly reported barriers to coproduction were within the domain of Capability. This was in relation to awareness of the concept of coproduction, concerns about the limited abilities of sections of society to be partners in coproducing health care and poor health literacy. Most participants were supportive of the concept of coproduction, once they were informed of its meaning, but awareness of coproduction as a guiding principle of the NHS in Wales was poor. Furthermore, when coproduction was discussed, it was usually in the form of patient involvement in decisions about their own care processes rather than involvement in the planning, delivery and improvement of the service as a whole.

Opportunities to engage in coproduction were seen to be limited by clinicians’ time and the resources available to them. However, many spoke positively about the role that third‐sector organizations, particularly health charities, can play in facilitating more informed and empowered citizens.

In terms of Motivation, some clinicians and public participants appeared to be expressing concerns about whether coproduction had the potential to improve health provision. This was expressed in terms of previous negative experiences of involvement (or of involving patients). Likewise, there were some doubts about whether it was feasible or sustainable for citizens to be empowered as equal partners within the current system.

### 4.2 Comparison with existing research

Our data confirm those of other studies, which illustrate that effective communication between practitioners and citizens can facilitate coproduction,^6^, ^22^, ^23^ particularly during consultations where patients and clinicians are engaged in shared decision making. Considerable work has already been conducted on the importance of heath literacy^24^ and patient empowerment^25^, ^26^ as a means to move away from paternalistic styles to more equitable and collaborative styles of health‐care delivery. The wide policy priority for patient‐centred care and shared decision making^27^ has encouraged professionals and patients to examine new ways of enabling participation.

Other authors have highlighted the social and economic barriers to coproduction,^28^, ^29^, ^30^ and these factors were also evident in our study. Conversely, the value of shared responsibility for outcomes is debatable and too much patient autonomy can result in poor outcomes and inefficiency in the system.^31^ However, the health service cannot abandon patients who do not have the capacity or personal resources to partner effectively.

Our findings also reflect the work of previous studies which demonstrate that citizen activation can be enhanced by developing the role that community organizations play in facilitating more informed and empowered citizens.^32^ While in our study this was seen as a positive move and a facilitator to coproduction, concerns have been expressed about the appropriation of a movement which may have genuinely started to give citizens and community groups more control, but has since been conflated with discourses of austerity and public service retrenchment.^33^ Finally, power sharing requires new forms of accountability and conceding of some professional control. Other authors have also noted that coproduction can bring with it a host of concerns such as competency and risks.^34^

### 4.3 Implications for policy and practice

Coproduction is an increasingly popular concept, and while often unfamiliar to clinicians and the public, once explained it is found to be broadly acceptable. Despite this, there is a need for further guidance and support from health services, partner organizations and governments to encourage a shared understanding among staff and members of the public about what coproduction might mean in the context of health care. Implementing coproduction in the NHS will also require strengthening the capability of staff and citizens to share power and responsibility in relation to health. This might involve the enhancement of physical and social opportunities, including staff training and partnerships with third‐sector organization.

### 4.4 Strengths and limitations

The COM‐B model offered clear methods to identify barriers and facilitators to coproduction within the wider context of Prudent Healthcare. In this study, we incorporated the views of public participants representing a variety of ages and socio‐economic backgrounds across Wales, although there is limited representation of employed public participants. Some clinical groups were also underrepresented, such as junior doctors, who could have added useful perspectives to the findings.

### 4.5 Further research

Future research could evaluate the feasibility, acceptability and effectiveness of implementing interventions to promote coproduction within the NHS. Interventions might include staff and undergraduate training and partnerships with third‐sector organizations.

## 5 CONCLUSIONS

Coproduction aims to improve the performance of existing public services by actively involving citizens in their design and evaluation. Our study highlights the varying yet broadly positive perceptions among the public and clinicians to coproduction in relation to health services. Despite a general lack of awareness of the term, many participants felt that the key elements of coproduction have been accepted and used in parts of clinical practice. Health policies designed to produce a shift to a more coproduced model of health‐care delivery should consider that actors (patients, health‐care providers and the health‐care system itself) will be constrained by some of the barriers identified in this study. These include lack of knowledge relating to how to conceptualize and undertake coproduction, attitudes of patients and clinicians towards power sharing and service‐level barriers such as the allocation of time for appointments. Importantly, if coproduction is to become a reality in health care and these barriers are to be mitigated, then training for staff within the NHS and partnership organizations is required to promote opportunities for understanding and implementing coproduction.

## CONFLICT OF INTERESTS

There are no conflict of interests.

## AUTHORS’ CONTRIBUTIONS

Fiona Wood was the Principal Investigator who led the application for funding and led the Study Management Group. Daniella Holland‐Hart and Samia Addis collected, coded and analysed the data. Daniella Holland‐Hart drafted the manuscript. All authors contributed to the recruitment of participants, discussion of data and review and editing of the transcript. All Authors read and approved the final manuscript.

## ETHICS

Ethical approval for the study was sought and granted on 2 December 2015 (ref: 15/YH/0545) by Yorkshire and The Humber—Leeds East Research Ethics Committee.
